# Addressing Loss of Efficiency Due to Misclassification Error in Enriched Clinical Trials for the Evaluation of Targeted Therapies Based on the Cox Proportional Hazards Model

**DOI:** 10.1371/journal.pone.0153525

**Published:** 2016-04-27

**Authors:** Chen-An Tsai, Kuan-Ting Lee, Jen-pei Liu

**Affiliations:** 1 Division of Biometry, Department of Agronomy, National Taiwan University, Taipei, Taiwan; 2 Institute of Epidemiology, National Taiwan University, Taipei, Taiwan; 3 Division of Biostatistics and Bioinformatics, Institute of Population Health Sciences, National Health Research Institutes, Zhunan, Taiwan; University Medicine Greifswald, GERMANY

## Abstract

A key feature of precision medicine is that it takes individual variability at the genetic or molecular level into account in determining the best treatment for patients diagnosed with diseases detected by recently developed novel biotechnologies. The enrichment design is an efficient design that enrolls only the patients testing positive for specific molecular targets and randomly assigns them for the targeted treatment or the concurrent control. However there is no diagnostic device with perfect accuracy and precision for detecting molecular targets. In particular, the positive predictive value (PPV) can be quite low for rare diseases with low prevalence. Under the enrichment design, some patients testing positive for specific molecular targets may not have the molecular targets. The efficacy of the targeted therapy may be underestimated in the patients that actually do have the molecular targets. To address the loss of efficiency due to misclassification error, we apply the discrete mixture modeling for time-to-event data proposed by Eng and Hanlon [8] to develop an inferential procedure, based on the Cox proportional hazard model, for treatment effects of the targeted treatment effect for the true-positive patients with the molecular targets. Our proposed procedure incorporates both inaccuracy of diagnostic devices and uncertainty of estimated accuracy measures. We employed the expectation-maximization algorithm in conjunction with the bootstrap technique for estimation of the hazard ratio and its estimated variance. We report the results of simulation studies which empirically investigated the performance of the proposed method. Our proposed method is illustrated by a numerical example.

## Introduction

**Fig 1:**
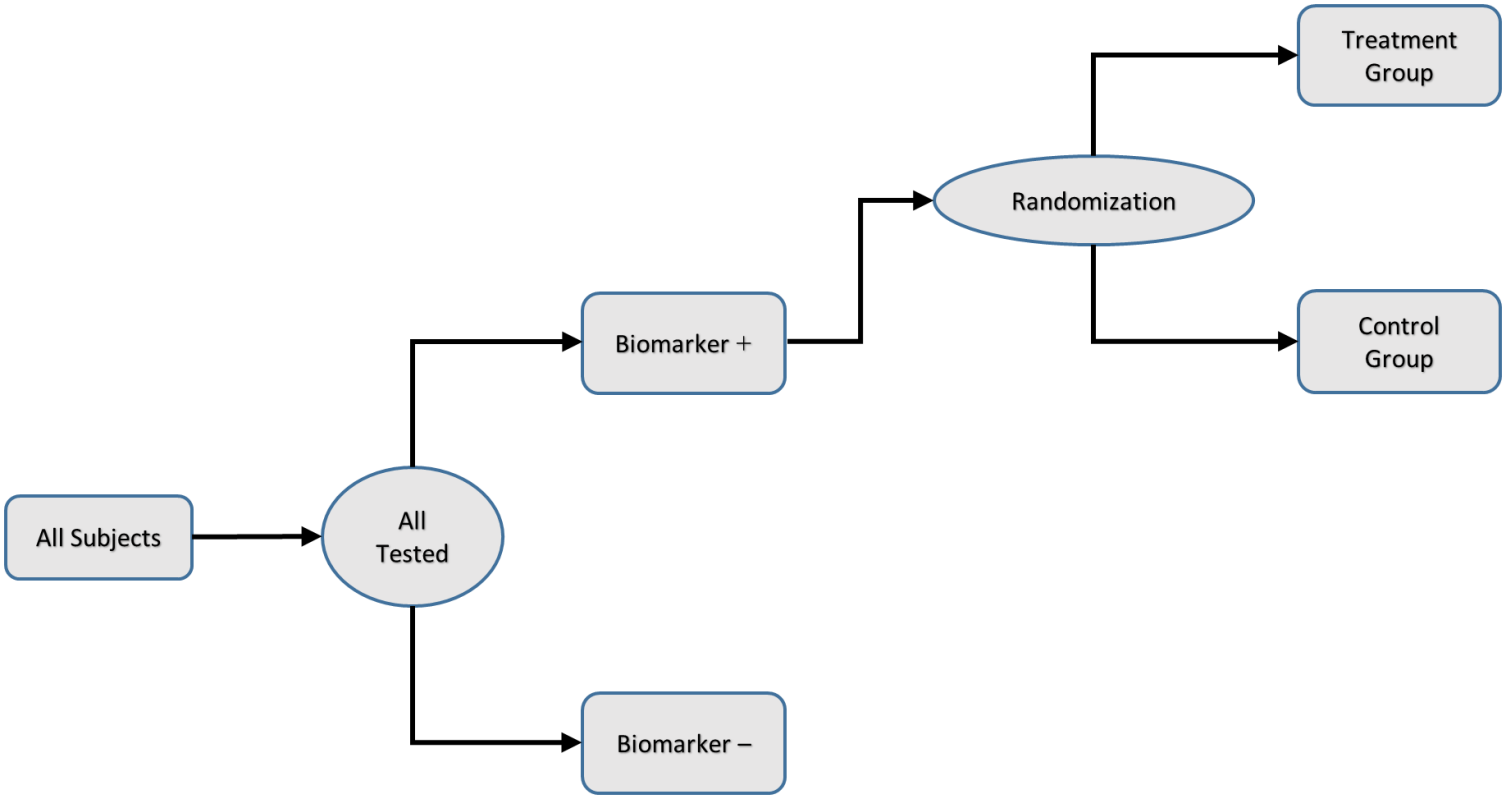
Randomization Schema of enrichment design for targeted clinical trials. *Source*: FDA [4].

On February 20, 2015, in his State of the Union Address, US President Obama announced the launching of a new Precision Medicine Initiative (PMI). As pointed out by Collins and Varmus [1], the near-term goal of the PMI is focused on cancers each of which has its own genomic signature driven by molecular lesions. In response to the PMI, starting in 2014, the US National Cancer Institute (NCI) launched a series of novel, molecularly guided trials which include the Exceptional Responders Initiative, NCI MATCH, ALCHEMIST trial and Master Protocol for second-line treatment of squamous lung cancer [2]. Most molecularly guided trials employ the enrichment design [3]. The enrichment design consists of two phases. The first phase is the enrichment phase during which each patient is tested for specified molecular targets using a diagnostic device that is capable of detecting them. Only those patients testing positive for the specified molecular targets are enrolled into the second phase in which they are randomly assigned either to the targeted treatment or to the concurrent control treatment. Fig 1 provides a graphical representation of the randomization schema of an enrichment design [4].

The enrichment design may be an efficient design for evaluation of targeted therapies. However, very few diagnostic devices have a perfect accuracy with 100% positive predictive value (PPV). In particular, the PPV can be very low for some rare diseases with low prevalence. Consequently, a sizable proportion of the patients tested positive during the enrichment phase may not actually have the molecular targets of interest. On the other hand, the targeted therapy is only designed to be efficacious for the patients actually having the molecular targets. The targeted therapy may not only be ineffective but also cause harmful adverse reactions in those patients who do not have the molecular targets despite testing positive for them. Liu, et al. [5] showed that the treatment effect estimated without consideration of misclassification error is underestimated for the true-positive patients with the molecular targets. Liu, et al. [5] and Liu and Lin [6] proposed inferential procedures, based on the expectation-maximization (EM) algorithm in conjunction with the bootstrap technique, to estimate the actual treatment effect of the targeted therapy for continuous and binary endpoints, respectively, under the enrichment design. For the censored endpoints, Chen, et al. [7] under assumption of a one-parameter exponential distribution, suggested a parametric method for the inference of the hazard ratio for the targeted therapy under the enrichment design.

Most of clinical trials for the near-term goal in the PMI are for evaluation of targeted therapies in treatment of various cancers with progression-free survival (PFS) or overall survival (OS) as the primary or secondary efficacy endpoints. The Cox proportional hazard (PH) model is the most commonly employed statistical method for inference of treatment effects which takes individual patient characteristics into account. Consequently, we propose an application of the discrete mixture modeling for time-to-event data proposed by Eng and Hanlon [8] to develop an inferential procedure based on the Cox PH model [9] for estimating the treatment effect of the targeted therapy for the true-positive patients with molecular targets. In our approach, we assume that the number of classes is two: true-positive patients and false-positive patients. Following Eng and Hanlon [8], we only assume that the hazards are proportional within each class. Combining the expectation-maximization (EM) algorithm with the bootstrap technique, we obtain the estimates of hazard ratios with their estimated standard errors. Because the assumption of a common proportional hazard is relaxed, we are able to obtain the estimated hazard ratio not only for the patients truly with the molecular targets but also for the patients truly without the molecular targets. Hence, we can perform the inference for target-by-treatment interaction.

In the next section, our proposed method for taking into consideration the diagnostic inaccuracy and uncertainty, based on discrete mixture modeling and the EM algorithm for the Cox PH model, is introduced with inferential procedures. The results of numerical studies, including numerical examples and simulation studies, are provided in the third section. Numerical examples illustrate the application of our proposed method in practical scenarios. Simulation results provide empirical performance of our proposed methods in terms of size, power and coverage probability of confidence intervals. Final remarks and discussion are presented in the last section.

## Methods

### Enrichment Design and Likelihood Function

We developed the proposed procedure for the following situations. First, a specified molecular target has been identified in the pathway of pathogenesis of the disease. Secondly, a validated diagnostic device is available for detection of the specified molecular target with a known estimated positive predicted value from validation studies. Third, this device is only for detection of the molecular target and is not prognostic for clinical outcomes of patients. A targeted therapy is currently being developed for true-positive patients with the molecular target. Finally, the enrichment design is employed to evaluate the treatment effect of the targeted therapy for the patients tested positive for the molecular target by the diagnostic device. Our objective is to develop statistical inference for the treatment effect of the targeted therapy for the true-positive patients with the specified molecular target.

Although under the enrichment design, all randomize patients have tested positive for the molecular target, due to inaccuracy of the diagnostic device, some positive patients still may not have the specified molecular target. Therefore, there are two latent classes of patients: the patients with the molecular target (true-positive patients) and the patients without the molecular target (false-positive patients). (Pepe [10]) By naïvely assuming no misclassification error, one can apply the Cox proportional hazards model for inference of treatment effect of the targeted therapy without adjustment for diagnostic inaccuracy. This approach is referred to as the naïve method.

For simplicity, we only consider one covariate Z for treatment identification in the Cox PH model. Z is 1 for the targeted therapy and 0 for the concurrent control. For the patients with the molecular target (+), let (*y*_+*i*_, *δ*_+*i*_, *z*_+*i*_), *i* = 1,…,*n*_+_ be an independent right-censored sample with right-censored survival time, y_+*i*_, covariates z_+*i*_, and censored indicator δ_+*i*_ (δ_+*i*_ = 1 if the event occurs; = 0 if censored). (*y*_−*i*_, *δ*_−*i*_, *z*_−*i*_) is similarly defined for the patients without the molecular target (-), *i* = 1,…,*n*_−_. Denote the collection of right-censored times, censored indicator and covariate for all patients as

$$Y=(y_{+i},\ldots ,y_{+n_{+}},y_{-i},\ldots y_{-n_{-}})\;,\;\Delta =(\delta_{+i},\ldots ,\delta_{+n_{+}},\delta_{-i},\ldots \delta_{-n_{-}})\;,\;Z=(z_{+i},\ldots ,z_{+n_{+}},z_{-i},\ldots z_{-n_{-}})$$

We further assume a Cox PH model separately for the patients with and without the molecular target. Hence, under the PH assumption, the hazard function for true target status h is given as

$$h_{hi}(t|z_{h})=h_{0h}(t)\text{exp}(\lambda_{h}z_{hi}),$$ (1)

where *h*_0*h*_(*t*) and *λ*_*h*_ are the baseline hazard and the treatment effect (regression coefficient: log hazard ratio) with treatment indicator *z*_*hi*_, respectively, for patient *i* with true target status *h*, for *i* = 1,…,*n*_*h*_;*h* = +,−.

Table 1 gives the baseline hazards and log hazard ratios between treatment groups for the true-positive and false-positive patients.

**Table 1:** Baseline hazards by diagnostic result of the molecular target.

Positive Diagnostic	True target condition	Accuracy of diagnosis	Baseline Hazard	Log hazard ratio between treatment
+	+	γ	h_0+_(y_+i_)	λ_+_
	-	1-γ	h_0-_(y_-i_)	λ_-_

γ is the positive predicted value

The hypothesis of interest for the true-positive patients with the molecular target is given as

$$H_{0}:\lambda_{+}=0\begin{array}{cc} & vs.\begin{array}{cc} & H_{a}:\lambda_{+}\neq 0 \end{array} \end{array}$$

The density function of the PH model for right censored survival times for true target status h is given as

$$f_{h}(y_{hi},\delta_{hi}|z_{hi};\lambda_{h})={\left[h_{0h}(y_{hi})\text{exp}(\lambda_{h}z_{hi})\right]}^{\delta_{hi}}\text{exp}\left\{-H_{0h}(y_{hi})\text{exp}(\lambda_{h}z_{hi})\right\},$$ (2)

where *H*_0*h*_(*y*_*hi*_) is the cumulative baseline hazard function for patient *i* with molecular status *h*,*i* = 1,…,*n*_*h*_;*h* = +,−.

To accommodate the inaccuracy of the diagnostic device and the variability of its estimate, we introduce a latent binary variable *X*_*i*_ for the true target status of patient *i* which is independently and identically distributed (i.i.d.) as a Bernoulli distribution with probability γ, where γ is the PPV of the diagnostic device, *i* = 1,…,N; N = n_+_ + n___. In other words, *X*_*i*_ = 1 if patient i has the molecular target; = 0 if patient *i* lacks the molecular target and P(*X*_*i*_ = 1) = γ = 1−P(*X*_*i*_ = 0), *i* = 1,…,N. The density function of (y_+*i*_, y_-*i*_) given latent variable *X*_*i*_ is given as

$$\begin{array}{l} f_{+,-}(y_{+},y_{-},\delta_{+},\delta_{-}|z_{+},z_{-},x_{i};\gamma ,\lambda_{+},\lambda_{-})\\ ={\left[f_{+}(y_{+i},\delta_{+i}|z_{+i};\lambda_{+})\right]}^{x_{i}}{\left[f_{-}(y_{-i},\delta_{-i}|z_{-i};\lambda_{-})\right]}^{1-x_{i}}\\ ={\left\{{\left[h_{0+}(y_{+i})\text{exp}(\lambda_{+}z_{+i})\right]}^{\delta_{+i}}\text{exp}\left[-H_{0+}\text{exp}(\lambda_{+}z_{+i})\right]\right\}}^{x_{i}}{\left\{{\left[h_{0-}(y_{-i})\text{exp}(\lambda_{-}z_{-i})\right]}^{\delta_{-i}}\text{exp}\left[-H_{0-}\text{exp}(\lambda_{-}z_{-i})\right]\right\}}^{1-x_{i}} \end{array}$$

Hence, the joint density function of (*y*_+*i*_, *y*_−*i*_, *x*_*i*_) is

$$\begin{array}{l} {\left\{\gamma {\left[h_{0+}(y_{+i})\text{exp}(\lambda_{+}z_{+i})\right]}^{\delta_{+i}}\text{exp}\left[-H_{0+}\text{exp}(\lambda_{+}z_{+i})\right]\right\}}^{x_{i}}\\ \times {\left\{(1-\gamma ){\left[h_{0-}(y_{-i})\text{exp}(\lambda_{-}z_{-i})\right]}^{\delta_{-i}}\text{exp}\left[-H_{0-}\text{exp}(\lambda_{-}z_{-i})\right]\right\}}^{1-x_{i}}. \end{array}$$ (3)

Let **ψ** = (*γ*, *h*, *λ*_+_, *λ*_−_) denote the vector of unknown parameters, where *h* = {*h*_0+_(*y*_+*i*_), *h*_0−_(*y*_−*i*_)} indicates the set of baseline hazard functions. It follows that the complete-data log-likelihood function for **ψ** is given as

$$\begin{array}{l} \text{ln}L_{C}(\Psi )=\text{ln}\gamma \sum_{i=1}^{N}x_{i}+\text{ln}(1-\gamma )\sum_{i=1}^{N}\left(1-x_{i}\right)\\ \;+\sum_{i=1}^{N}\left[\delta_{+i}x_{i}\text{ln}(h_{0+})+\delta_{\text{+}i}x_{i}z_{+i}\lambda_{+}-x_{i}H_{0+}\text{exp}(\lambda_{+}z_{+i})\right]\;\\ \;+\sum_{i=1}^{N}\left[\delta_{-i}(1-x_{i})\text{ln}(h_{0-})+\delta_{-i}(1-x_{i})z_{-i}\lambda_{-}-(1-x_{i})H_{0-}\text{exp}(\lambda_{-}z_{-i})\right] \end{array}$$ (4)

### Application of Discrete Mixture Modeling and the EM Algorithm

To obtain the maximum likelihood estimates of the treatment effect by the expectation-maximization (EM) algorithm, in the E-step, we need to find the conditional expectation of *X*_i_ given *Y*_+*i*_, *Y*_*−i*_, *δ*_+*i*_, *δ*_−*i*_, *Z*_+*i*_, *Z*_−*i*_; *γ*, *h*, *λ*_+_, *λ*_−_. Denoting the current estimates of the parameters after *k* iterations as ${\hat{\gamma }}^{\left(k\right)},{\hat{h}}_{0h}^{\left(k\right)},{\hat{H}}_{0h}^{\left(k\right)},{\hat{\lambda }}_{h}^{\left(k\right)}$ and letting

$$A_{i}={\hat{\gamma }}^{\left(k\right)}{\left[{\hat{h}}_{0+}^{\left(k\right)}(y_{+i})\text{exp}({\hat{\lambda }}_{+}^{\left(k\right)}z_{+i})\right]}^{\delta_{+i}}\text{exp}\left[-{\hat{H}}_{0+}^{\left(k\right)}\text{exp}({\hat{\lambda }}_{+}^{\left(k\right)}z_{+i})\right]$$

and

$$B_{i}=(1-{\hat{\gamma }}^{\left(k\right)}){\left[{\hat{h}}_{0-}^{\left(k\right)}(y_{-i})\text{exp}({\hat{\lambda }}_{-}^{\left(k\right)}z_{-i})\right]}^{\delta_{-i}}\text{exp}\left[-{\hat{H}}_{0-}^{\left(k\right)}\text{exp}({\hat{\lambda }}_{-}^{\left(k\right)}z_{-i})\right]$$

it follows that in the E-step, the conditional expectation is given as

$$\begin{array}{lll} x_{i}^{\left(k\right)}=E_{\Psi (K)}(X_{i}|Y_{+i},Y_{-i},\delta_{+i},\delta_{-i};\gamma ,h,\lambda_{+},\lambda_{-}) & = & P(x_{i}=1|y_{+i},y_{-i},\delta_{+i},\delta_{-i};\gamma ,h,\lambda_{+},\lambda_{-})\\ & = & \frac{A_{i}}{A_{i}+B_{i}}. \end{array}$$ (5)

In the M-step, the updated positive predicted value (PPV) *γ* is given as

$${\hat{\gamma }}^{\left(k+1\right)}=\frac{\sum_{i=1}^{N}{\hat{x}}_{i}^{\left(k\right)}}{N}.$$ (6)

Following Eng and Hanlon [8], the estimates of the baseline and cumulative baseline hazard functions as a function of hazard ratios are given below respectively:

$$\begin{array}{l} {\hat{h}}_{0+}^{\left(k+1\right)}(Y_{i})=\frac{{\hat{x}}_{i}^{\left(k\right)}}{\sum_{I:Y_{j}\leq Y}{\hat{x}}_{j}^{\left(k\right)}\text{exp}({\hat{\lambda }}_{+j}^{\left(k\right)}z_{+j})}\\ {\hat{h}}_{0-}^{\left(k+1\right)}(Y_{i})=\frac{1-{\hat{x}}_{i}^{\left(k\right)}}{\sum_{I:Y_{j}\leq Y}\left(1-{\hat{x}}_{j}^{\left(k\right)})\text{exp}({\hat{\lambda }}_{-j}^{\left(k\right)}z_{-j}\right)}\\ {\hat{H}}_{0+}^{\left(k+1\right)}(Y_{i})=\sum_{I:Y_{j}\leq Y_{i}}\frac{{\hat{x}}_{i}^{\left(k\right)}}{\sum_{I:Y_{j}\leq Y}{\hat{x}}_{j}^{\left(k\right)}\text{exp}({\hat{\lambda }}_{+j}^{\left(k\right)}z_{+j})}\\ {\hat{H}}_{0-}^{\left(k+1\right)}(Y_{i})=\sum_{Y_{j}\leq Y_{i}}\frac{1-{\hat{x}}_{i}^{\left(k\right)}}{\sum_{I:Y_{j}\leq Y}\left(1-{\hat{x}}_{j}^{\left(k\right)})\text{exp}({\hat{\lambda }}_{-j}^{\left(k\right)}z_{-j}\right)}. \end{array}$$ (7)

The estimation procedure of the EM-based approach is summarized as follows and then explained more fully step-by-step:

1. Set initial values for *γ*^(0)^,$\lambda_{+}^{\left(0\right)}$,$\lambda_{-}^{\left(0\right)}$,$h_{0+}^{\left(0\right)}$, $h_{0-}^{\left(0\right)}$.
2. Calculate *E*_*ψ*(*K*)_{*X*_*i*_|*Y*_+*i*_, *Y*_−*i*_, *δ*_+*i*_, *δ*_−*i*_, *Z*_+*i*_, *Z*_−*i*_;*γ*,*h*,*λ*_+_,*λ*_−_} using Eq (5), update *h*_0+_, *h*_0−_, *λ*_+_, *λ*_*−*_ by Eq (7), and update γ by Eq (6).
3. Repeat Step 2 until convergence.

For the initial values, the estimated PPV from the validation studies can be used as the initial value of *γ*^(0)^. $\lambda_{-}^{0}$ can be set as 1 and $\lambda_{+}^{0}$ is the hazard ratio specified in the protocol of the enrichment design. The standard error of the log hazard ratio for the true-positive patients with the molecular target is estimated by bootstrap procedures [11].

Step 1. Choose a large bootstrap sample size B. We would suggest B ≥ 1000. For 1 ≤ b ≤ B, generate the bootstrap samples $\left(y_{obs}^{b},\delta_{obs}^{b},x_{obs}^{b}\right)$, according to the probability model in Eq (4). The parameters employed to generate bootstrap sample $\left(y_{obs}^{b},\delta_{obs}^{b},x_{obs}^{b}\right)$ are replaced by the estimators obtained from the EM algorithm based on the original observations from the targeted clinical trial.
Step 2. Estimates ${\hat{\lambda }}_{+b}^{*}$ are obtained by applying the EM algorithm to the bootstrap sample $\left(y_{obs}^{b},\delta_{obs}^{b},x_{obs}^{b}\right)$, b = 1,…,B.
Step 3. An estimator of the variance of ${\hat{\lambda }}_{+}$ is given as

$$S_{+B}^{2}=\;\frac{\sum_{b=1}^{B}{\left({\hat{\lambda }}_{+b}^{*}-{\bar{\hat{\lambda }}}_{+}^{*}\right)}^{2}}{B-1},$$ (8)

where

$${\bar{\hat{\lambda }}}_{+}^{*}\;=\;\frac{\sum_{b=1}^{B}{\hat{\lambda }}_{+b}^{*}}{B}.$$

The null hypothesis is rejected and the efficacy of the targeted therapy is found to be different from that of the control group for the true-positive patient population at the α significance level if

$$z\;=\;\left|\frac{{\hat{\lambda }}_{+}}{\sqrt{S_{+B}^{2}}}\right|\;\geq \;z_{\alpha /2},$$ (9)

where z_α/2_ is the α/2 upper percentile of a standard normal distribution, and ${\text{S}}_{\text{+B}}^{2}$ denotes the estimated variance of ${\hat{\lambda }}_{+}$ obtained by the bootstrap procedure. The corresponding 100(1−α)% asymptotic confidence interval for λ_+_ can be constructed as ${\hat{\lambda }}_{+}\pm z_{\alpha /2}\sqrt{S_{+B}^{2}}.$ Hence the 100(1−α)% asymptotic confidence interval of the hazard ratio for the true-positive patients with the molecular target is given as $\text{exp}[{\hat{\lambda }}_{+}\pm z_{\alpha /2}\sqrt{S_{+B}^{2}}].$

### Simulation Setup

We conducted extensive simulation studies to empirically investigate and compare performance of our proposed method with the current procedure in terms of relative bias, coverage probability of confidence intervals, size and power. A modified R function by Eng and Hanlon [8] was employed in the simulation studies. Random samples of patient units with or without the molecular target were generated from the Bernoulli distribution with probability γ. Then the units were randomized in a 1:1 ratio to the targeted therapy group or concurrent placebo-control group. Although we used a semi-parametric model which does not require the actual distribution, we still need the distributional form for the data used in the simulation studies. We generated the one-parameter exponential random variable data with the specified parameters λ_+_, λ_−_ according to the status of the molecular target. It is presumed that the placebo control is not efficacious in the patients either with or without the molecular target. In addition, the targeted therapy is presumed ineffective in the false-positive patients without the target. The pair of survival times and censored indicators (y, δ) was generated according to the methods described in Chen, et al. [7].

The following specifications of parameters were considered in the simulation studies. The PPV was set to be 0.5, 0.6, 0.7, 0.8, and 0.9, which reflect a range of low to high positive predicted value. The censored proportions considered in the simulation studies were 0, 0.1, 0.2, 0.3 and 0.4. To investigate the finite sample properties, the sample sizes were set as 300, 600, and 900 per group. The size was evaluated at the hazard ratio of 1. The power of the proposed procedure was investigated at hazard ratios of 0.70, 0.75, 0.80 and 0.85. For each of 300 combinations, 1000 random samples were generated and the number of the bootstrap samples was also set to be 1000.

For estimation, we investigated the bias of the estimators and the coverage probability of the 95% confidence interval. For hypothesis testing, the performance measures included empirical size and power. The bias was estimated as the average of the differences between the estimates and the true value of the hazard ratio over 1000 simulated samples. The coverage probability was calculated as the proportion of the 1000 95% confidence intervals that contain the true value of the hazard ratio. The size and power were computed as the proportion of the 1000 samples for which the null hypothesis (H_0_: λ_+_ = 0 vs. H_a_: λ_+_ ≠ 0) was rejected for the two-sided test at the 5% significance level. For a 95% confidence level, a simulation study with 1000 simulated random samples implies that 95% of the empirical coverage probabilities of all combinations would be within 0.9365 and 0.9635 if the proposed method provides sufficient coverage probability. In addition, for the 5% nominal significance level, a simulation study with 1000 random samples implies that 95% of the empirical sizes would be within 0.0365 and 0.0635 if the proposed method can adequately control the size at the nominal level of 0.05.

## Results

### Numerical Examples

We constructed a dataset for a hypothetical scenario based on the information provided by the US Food and Drug Administration (FDA) in the package insert of Herceptin^®^[12]. A targeted therapy is being developed for the treatment of patients with a certain cancer whose specific molecular target is over-expressed as measured by an immunohistochemical (IHC) assay. Suppose that the IHC assay has a PPV of 0.75. From previous studies, the hazard ratios for the patients truly with and without the target are 0.7 and 1.26, respectively. Under the enrichment design, 480 patients with positive test results were randomized in 1:1 ratio to receive the targeted therapy plus the standard chemotherapy or to the standard chemotherapy alone. The censored rate is assumed to be 30%. Table 2 provides the point estimates of the hazard ratio between the two groups, their standard error, and 95% confidence intervals. In addition, Breslow’s estimates of baseline hazards were computed for the true-positive and false-positive patients when the PPV was set at 0.75 [13]. The baseline hazards of the true-positive and false-positive patients were 0.0027 and 0.008 respectively. Therefore, the baseline hazard of the true-positive patients was lower than that of the false-positive patients.

**Table 2:** Point and interval estimators of hazard ratios.

Results	Naive	EM (PPV = 0.75)
Hazard ratio	0.8318	0.7026
S.E. of log hazard ratio	0.1152	0.1439
95% L.C.I.	0.6637	0.5299
95% U.C.I.	1.0425	0.9315

L.C.I.: Lower confidence interval

U.C.I.: Upper confidence interval

When the PPV was 0.75, the naive approach without consideration of inaccuracy of diagnostic device and the variability of its estimate yields the estimate of hazard ratio for mortality of 0.8318 with a 95% CI from 0.6637 to 1.0425. Because the 95% CI does contain 1, the observed hazard ratio of death is not statistically significantly different from 1 at the 5% significance level. The targeted therapy therefore fails to prove its superior efficacy over chemotherapy alone. On the other hand, our proposed EM method resulted in an estimated hazard ratio of 0.7026. The 95% CI for the hazard ratio is (0.5299, 0.9315), which does not contain 1. As a result, the efficacy of the targeted therapy plus chemotherapy is concluded superior to chemotherapy alone for the true-positive patients with the molecular target at the 5% significance level. Because 25% of the patients do not have the specified molecular target, failure to take into consideration inaccuracy of the diagnostic device leads to underestimation of the hazard ratio based on the naive method by a magnitude of 18.39%.

### Results of the Simulation Studies

**Fig 2:**
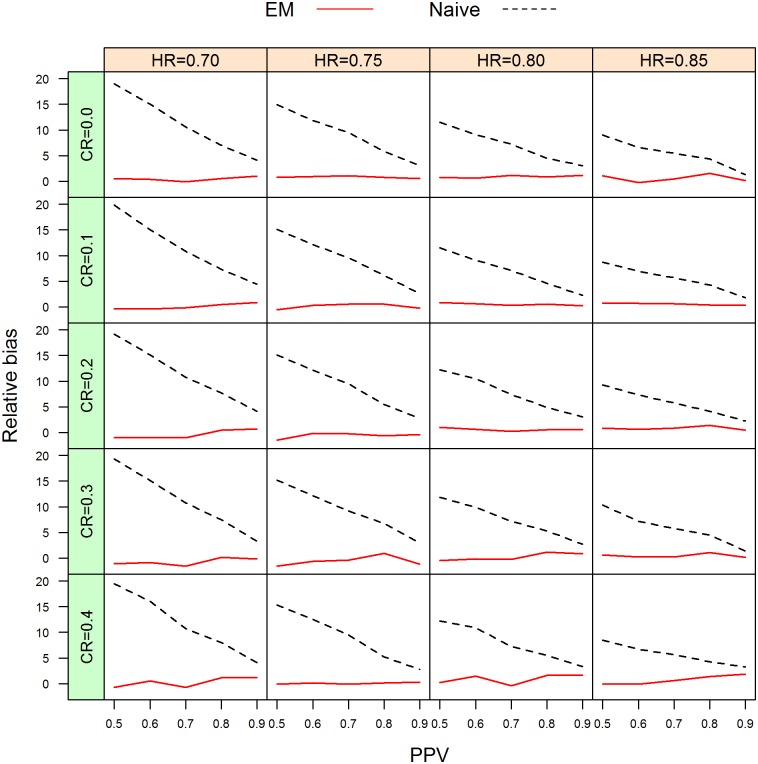
Relative bias curves between the proposed EM and naive approach for different censored rates (CR) at sample size n = 300 per group. Black line: naive; red line: proposed EM.

**Fig 3:**
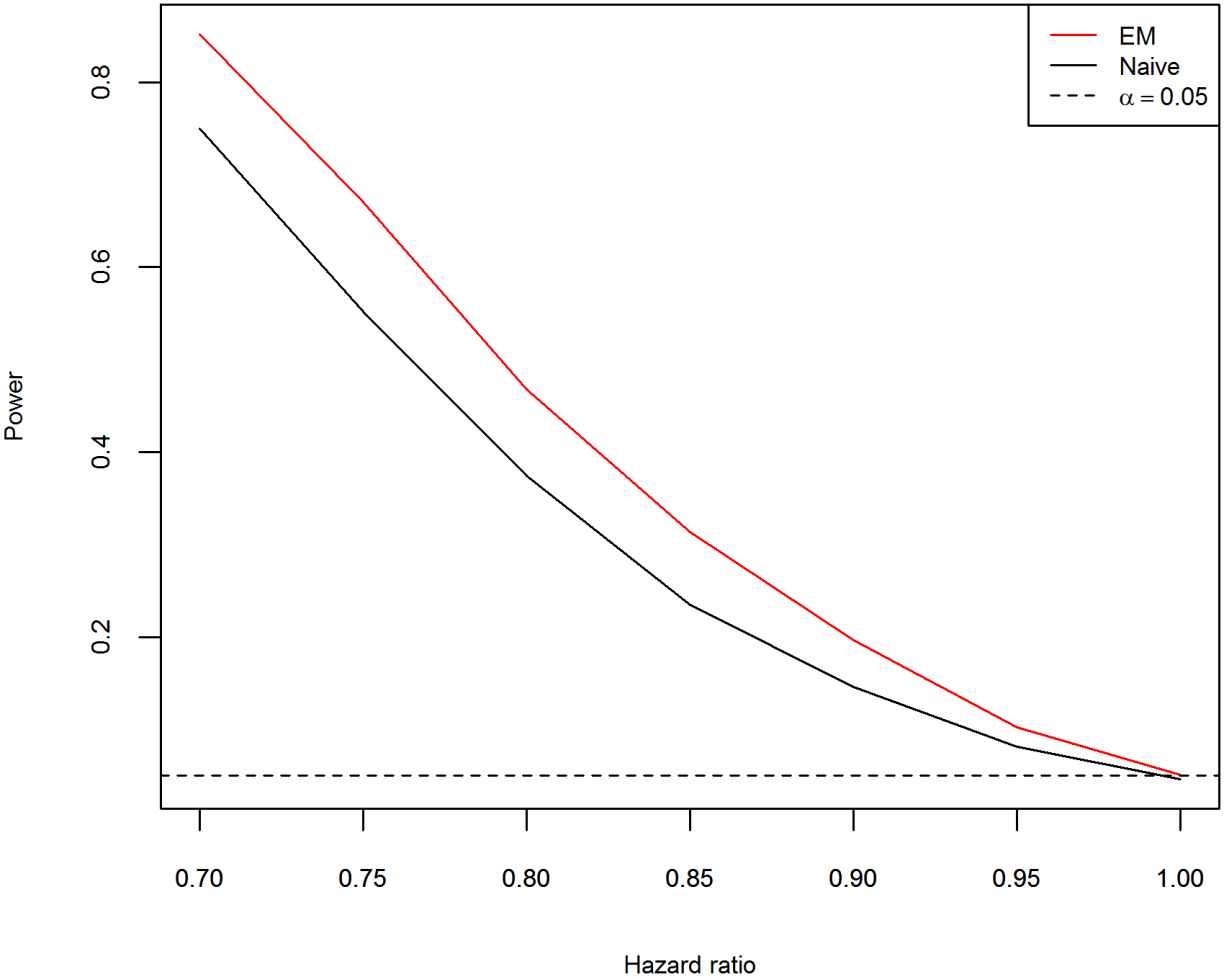
Empirical power curves when the PPV is 0.6, n = 300 per group and censored rate = 10%.

**Fig 4:**
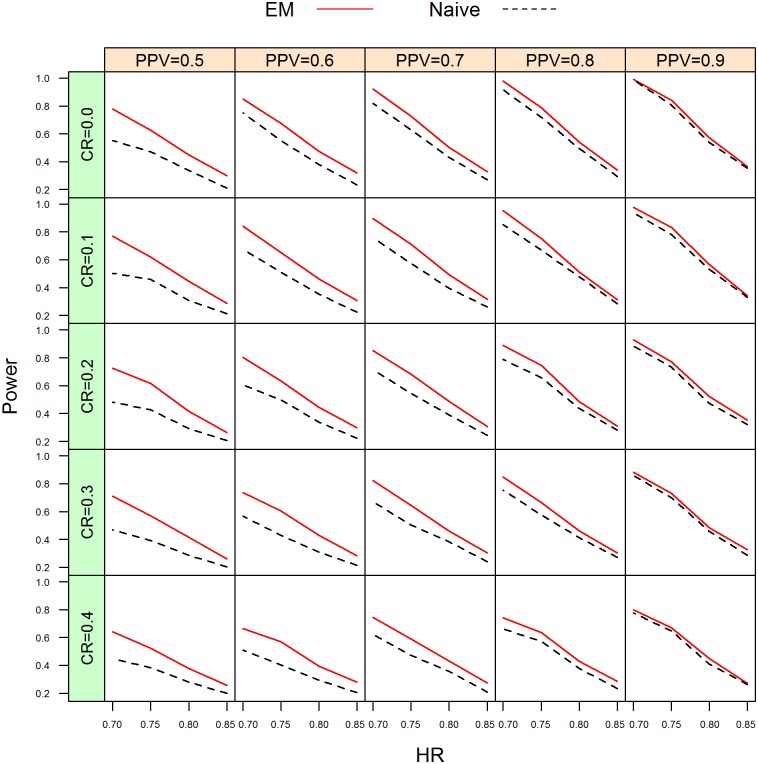
Empirical power curves between the proposed EM and naive approach for different censored rates (CR) at sample size n = 300 per group. Black line: naive; red line: proposed EM.

The simulation studies provide empirical results of performance of our proposed method compared with the current approach with respect to relative bias, coverage probability, size, and power. Due to the large volume of results generated in the simulation studies, we only present the results concerning relative bias, coverage probability and empirical power for the combinations with sample size of 300. They are provided in Table 3, Fig 2 and Table 4, Figs 3 and 4, respectively. All other results are provided in the Supporting Information.

**Table 3:** Relative bias (%) and coverage probability for n = 300 per group.

			PPV									
			0.5 (0.495^c^)		0.6 (0.601^c^)		0.7 (0.697^c^)		0.8 (0.796^c^)		0.9 (0.897^c^)	
N	HR	CR	Naive	EM	Naive	EM	Naive	EM	Naive	EM	Naive	EM
300	0.85	0	9.06^a^	1.06	6.59	-0.24	5.41	0.47	4.35	1.53	1.29	0.12
			0.889^b^	0.963	0.927	0.964	0.928	0.960	0.927	0.958	0.943	0.968
		0.1	8.71	0.78	6.92	0.72	5.71	0.62	4.24	0.36	1.79	0.32
			0.908	0.950	0.926	0.947	0.946	0.968	0.941	0.953	0.942	0.958
		0.2	9.29	0.82	7.29	0.59	5.76	0.82	4.12	1.41	2.24	0.47
			0.913	0.935	0.921	0.955	0.939	0.951	0.925	0.948	0.935	0.955
		0.3	10.35	0.59	7.18	0.24	5.76	0.24	4.47	1.06	1.41	0.12
			0.910	0.935	0.919	0.941	0.927	0.944	0.933	0.956	0.933	0.956
		0.4	8.47	-0.12	6.71	-0.12	5.65	0.59	4.24	1.41	3.29	1.88
			0.905	0.932	0.914	0.938	0.935	0.954	0.937	0.963	0.949	0.971
	0.8	0	11.50	0.75	9.12	0.63	7.25	1.13	4.50	0.88	3.00	1.13
			0.847	0.959	0.891	0.951	0.919	0.961	0.934	0.963	0.941	0.963
		0.1	11.50	0.13	9.12	0.13	7.12	0.01	4.62	0.50	2.25	0.25
			0.856	0.946	0.875	0.967	0.921	0.956	0.932	0.958	0.938	0.959
		0.2	12.25	1.00	10.50	0.63	7.37	0.25	4.87	0.50	3.00	0.63
			0.865	0.938	0.879	0.941	0.901	0.936	0.928	0.945	0.938	0.954
		0.3	11.88	-0.50	9.87	-0.13	7.12	-0.25	5.25	1.13	2.75	0.88
			0.878	0.937	0.901	0.944	0.921	0.943	0.924	0.942	0.933	0.948
		0.4	12.25	0.25	10.88	1.50	7.25	-0.38	5.50	1.62	3.37	1.62
			0.881	0.938	0.911	0.931	0.919	0.942	0.913	0.948	0.941	0.955
300	0.75	0	14.93	0.80	11.87	0.93	9.47	1.07	5.87	0.80	3.07	0.53
			0.778	0.962	0.856	0.954	0.892	0.965	0.922	0.964	0.932	0.956
		0.1	15.07	-0.53	12.13	0.27	9.60	0.53	6.13	0.53	2.53	-0.27
			0.799	0.946	0.869	0.951	0.907	0.958	0.931	0.959	0.941	0.963
		0.2	15.07	-1.47	12.13	-0.13	9.47	-0.27	5.47	-0.67	2.80	-0.40
			0.821	0.941	0.871	0.935	0.898	0.948	0.932	0.958	0.931	0.951
		0.3	15.20	-1.60	12.13	-0.67	9.20	-0.40	6.80	0.93	2.93	-1.20
			0.837	0.936	0.886	0.933	0.909	0.946	0.934	0.952	0.926	0.951
		0.4	15.33	-0.13	12.53	0.13	9.47	-0.13	5.20	0.13	2.80	0.27
			0.859	0.931	0.886	0.939	0.906	0.936	0.949	0.966	0.919	0.952
	0.7	0	19.00	1.57	15.00	0.71	10.57	0.57	7.00	0.57	4.14	1.00
			0.703	0.947	0.782	0.951	0.877	0.961	0.913	0.949	0.933	0.951
		0.1	19.86	-0.29	15.00	-0.43	10.86	-0.14	7.29	0.43	4.43	0.86
			0.726	0.951	0.799	0.942	0.881	0.948	0.919	0.952	0.941	0.963
		0.2	19.14	-1.00	15.14	-1.00	10.71	-1.00	7.71	0.43	4.14	0.71
			0.752	0.942	0.824	0.941	0.889	0.947	0.919	0.943	0.931	0.946
		0.3	19.29	-1.14	15.14	-0.86	10.71	-1.57	7.43	0.14	3.29	-0.14
			0.777	0.941	0.827	0.944	0.891	0.943	0.917	0.933	0.955	0.974
		0.4	19.43	-0.71	16.00	0.57	10.71	-0.71	8.00	1.14	4.14	1.14
			0.791	0.946	0.825	0.946	0.901	0.942	0.941	0.955	0.929	0.952

^a^: Relative bias (%)

^b^: Coverage probability

^c^: Estimate of PPV

CR: censored rate; HR: hazard ratio

**Table 4:** Comparison of empirical powers for n = 300 per group.

			PPV									
			0.5		0.6		0.7		0.8		0.9	
n	HR	CR	Naive	EM	Naive	EM	Naive	EM	Naive	EM	Naive	EM
300	0.85	0	0.211	0.298	0.234	0.318	0.269	0.328	0.292	0.338	0.353	0.366
		0.1	0.211	0.287	0.223	0.308	0.261	0.316	0.284	0.314	0.329	0.342
		0.2	0.207	0.263	0.224	0.298	0.242	0.306	0.282	0.309	0.320	0.351
		0.3	0.203	0.261	0.215	0.284	0.241	0.303	0.272	0.302	0.286	0.327
		0.4	0.198	0.256	0.205	0.279	0.207	0.274	0.233	0.284	0.263	0.272
	0.8	0	0.337	0.448	0.379	0.475	0.432	0.503	0.493	0.541	0.538	0.573
		0.1	0.307	0.445	0.352	0.462	0.395	0.494	0.477	0.511	0.531	0.565
		0.2	0.291	0.416	0.337	0.445	0.389	0.486	0.436	0.484	0.473	0.521
		0.3	0.287	0.415	0.308	0.429	0.384	0.461	0.413	0.462	0.459	0.483
		0.4	0.278	0.377	0.293	0.395	0.358	0.432	0.376	0.429	0.409	0.448
	0.75	0	0.472	0.626	0.553	0.674	0.630	0.727	0.718	0.786	0.808	0.841
		0.1	0.458	0.619	0.512	0.652	0.575	0.711	0.669	0.752	0.781	0.832
		0.2	0.426	0.616	0.498	0.633	0.544	0.683	0.656	0.747	0.735	0.773
		0.3	0.392	0.571	0.429	0.605	0.503	0.646	0.576	0.664	0.698	0.731
		0.4	0.382	0.524	0.403	0.569	0.473	0.589	0.572	0.636	0.646	0.671
	0.7	0	0.551	0.778	0.751	0.851	0.819	0.921	0.916	0.981	0.989	0.991
		0.1	0.502	0.769	0.675	0.842	0.757	0.896	0.852	0.953	0.939	0.976
		0.2	0.481	0.727	0.605	0.804	0.713	0.851	0.789	0.889	0.883	0.931
		0.3	0.469	0.711	0.566	0.736	0.671	0.823	0.754	0.848	0.859	0.882
		0.4	0.449	0.642	0.508	0.665	0.621	0.744	0.661	0.742	0.777	0.798

CR: censored rate; HR: hazard ratio

The simulation results show that the empirical estimates of the positive predicted values are close to the specified values for all the different combinations, with a maximal absolute difference of only 0.008. The simulation results on relative bias and coverage probabilities are given in Table 3 and S1 and S2 Tables. Graphical presentations of a summarization of Table 3, S1 and S2 Tables are given in Fig 2, S1 and S2 Figs, respectively. The absolute relative bias of the estimator of the hazard ratio for the true-positive patients obtained by the naive approach ranges from 1.27% to 19.84%. In comparison, the absolute relative bias of the estimator of the hazard ratio for the true-positive patients with the molecular target obtained by the EM algorithm is smaller than 2%. As PPV increases, the relative bias decreases. Overall, the relative bias is a decreasing function of the hazard ratio. There is no apparent relationship between relative bias and sample size and between relative bias and censoring proportion.

The results concerning the empirical coverage probabilities of the 95% confidence intervals for the hazard ratios by the current method and the EM method are described below. Only 20 of the 300 coverage probabilities (6.67%) of the 95% confidence intervals by the naive method exceed 0.9365. The coverage probability by the naive method are as low as 0.546. On the other hand, there are 245 of 300 coverage probabilities (81.7%) of the 95% confidence intervals by the EM method exceeding 0.9365. However, 280 of the 300 coverage probabilities (93.3%) of 95% confidence intervals constructed by the EM algorithm are above 0.93. No coverage probability of the EM method is below 0.91. The coverage probability is an increasing function of the PPV and a decreasing function of the hazard ratio and sample size. In summary, the proposed EM procedure not only gives nearly unbiased estimated treatment effect for the true-positive patients with the molecular target but also provides sufficient coverage probability.

The simulation results on the sizes are given in Table 5. From Table 5, the empirical sizes for the naive method and EM method for all combinations are within 0.0365 to 0.0635. The results demonstrate that both the naive and EM method can adequately control the size at the nominal level of 5%. The results concerning the empirical power for the naive method and EM method are given in Table 4, S3 and S4 Tables, respectively. Graphical presentations of a summarization of Table 4, S3 and S4 Tables are given in Fig 4, S3 and S4 Figs, respectively. In addition, Fig 3 presents the power curves when n = 300 per group, censored rate is 10%, and the PPV is 0.6. In is clear from Table 2, S3 and S4 Tables, that the empirical power is an increasing function of the PPV and a decreasing function of the censoring rate and the hazard ratio. For both methods, the power increases as the sample size increases. The simulation results of the empirical power demonstrate that the proposed EM procedure is uniformly more powerful than the naive method. In summary, under the PH model the proposed EM procedure not only can better control the size at its nominal level but also is more powerful than the naive method.

**Table 5:** Comparison of empirical sizes.

						PPV					
		0.5		0.6		0.7		0.8		0.9	
n	CR	Naive	EM	Naive	EM	Naive	EM	Naive	EM	Naive	EM
300	0	0.048	0.048	0.048	0.047	0.042	0.041	0.045	0.044	0.042	0.046
	0.1	0.053	0.049	0.051	0.048	0.053	0.045	0.049	0.046	0.041	0.041
	0.2	0.062	0.054	0.061	0.055	0.055	0.048	0.050	0.048	0.054	0.051
	0.3	0.062	0.057	0.061	0.058	0.062	0.051	0.053	0.047	0.058	0.049
	0.4	0.063	0.059	0.064	0.063	0.061	0.052	0.048	0.051	0.051	0.043
600	0	0.054	0.054	0.050	0.049	0.049	0.054	0.048	0.050	0.051	0.049
	0.1	0.051	0.048	0.053	0.047	0.052	0.056	0.055	0.054	0.052	0.053
	0.2	0.056	0.052	0.048	0.045	0.050	0.055	0.054	0.051	0.051	0.052
	0.3	0.053	0.048	0.051	0.050	0.056	0.051	0.051	0.050	0.052	0.052
	0.4	0.058	0.049	0.050	0.050	0.055	0.053	0.050	0.050	0.050	0.048
900	0	0.051	0.057	0.048	0.054	0.051	0.049	0.050	0.048	0.053	0.053
	0.1	0.054	0.055	0.049	0.047	0.050	0.051	0.052	0.049	0.048	0.048
	0.2	0.051	0.056	0.055	0.054	0.053	0.053	0.055	0.055	0.057	0.057
	0.3	0.052	0.055	0.048	0.056	0.052	0.052	0.049	0.049	0.051	0.050
	0.4	0.061	0.060	0.048	0.055	0.049	0.050	0.052	0.053	0.056	0.056

CR: censored rate

## Discussion

Although all patients enrolled into the enrichment design are tested positive for the specified molecular target, some of them may not actually have the target due to inaccuracy of the diagnostic device. The proportion of false-positive patients may be sizable for rare diseases with low prevalence rates. Consequently, the treatment effect of the targeted therapy may be severely underestimated for the true-positive patients with the molecular target. In addition, the magnitude of underestimation is a decreasing function of the PPV. The Cox PH model is the most frequently employed method for evaluation of targeted therapies for cancer trials with progression-free survival (PFS) or overall survival (OS) as the primary efficacy endpoint. We applied the method of discrete mixture modeling proposed by Eng and Hanlon [8], based on the Cox PH model, to develop an inferential procedure of estimating the treatment effect for the true-positive patients with the molecular target. Our proposed procedure employs the EM algorithm in conjunction with the bootstrap method not only to accommodate inaccuracy of the diagnostic device but also to take into consideration the variability of the estimated accuracy measure. Empirical evidence from extensive simulation studies demonstrates that our proposed method is nearly unbiased and provides sufficient coverage probability for the unknown hazard ratio for the true-positive patients with the molecular target. In addition, our suggested method can adequately control type I error at the pre-specified nominal level and is also uniformly more powerful than the naive method.

Because of the discrete mixture modeling, the PH assumption is relaxed such that the hazards are only assumed to be proportional within each group of patients either with or without the molecular target. Since the PPV is not 100%, the log hazard ratio for the patients without the molecular target can be similarly estimated by the EM algorithm. Its estimated standard error can be also obtained by the bootstrap method. Inference regarding the efficacy of the target therapy can similarly be made to the patients without the molecular target. In addition, the patients with the molecular target are independent of the patients without the target. We can also make inferences about the target-by-treatment interaction, i.e., the difference in the treatment effect between the groups of patients with and without the molecular target. Denote ${\hat{\lambda }}_{-}{\text{ and S}}_{\text{-B}}^{2}$ as the estimated log hazard ratio and its estimated variance for the patients without the molecular target. A (1-α)100% asymptotic confidence interval for *λ*_+_−*λ*_−_ is given by

$$({\hat{\lambda }}_{+}-{\hat{\lambda }}_{-})\pm z_{\alpha /2}\sqrt{S_{\text{+B}}^{2}+S_{\text{-B}}^{2}}.$$ (10)

A confidence interval for *λ*_+_−*λ*_−_ not only can be used to make inferences about the existence of target-by-treatment interaction but also can test whether the efficacy of the targeted therapy of the patients without the target is either equivalent or non-inferior to that of the patients with the molecular target.

Using the same dataset used in the Numerical Example, the estimated log hazard ratio for the patients without the molecular target is 0.259 with an estimated standard error of 0.3049. The corresponding estimated hazard ratio is 1.2956 with a 95% asymptotic confidence interval of (0.7179, 2.3551). It follows that a 95% asymptotic confidence for *λ*_+_ − *λ*_−_ is (-1.2728, 0.0049) which includes 0. The target-by-treatment interaction is, therefore, not statistically significant at the 0.05 significance level. However, the target-by-treatment interaction is significant at the 0.10 level because the 90% asymptotic confidence interval is (-1.1667, -0.0574), which does not include 0. Therefore, this analysis of the target-by-treatment interaction further demonstrates that even under the enrichment design our proposed procedure can evaluate whether the new targeted therapy is efficacious for false-positive patients if the PPV is not too high.

Our proposed method only considers one covariate in the PH model, which is the treatment indicator. However, we can also incorporate other covariates such as gender, age, or disease status in the model. The full likelihood with more than one covariate is given as

$$\begin{array}{lll} L_{C}(\psi ) & = & {\prod_{i=1}^{n}\left\{\gamma {\left[h_{0+}(y_{+i})\text{exp}(\lambda_{+}z_{+i}+\sum_{j=2}^{k}\beta_{+j}z_{+j})\right]}^{\delta_{+i}}\text{exp}\left[-H_{0+}\text{exp}(\lambda_{+}z_{+i}+\sum_{j=2}^{k}\beta_{+j}z_{+j})\right]\right\}}^{x_{i}}\\ & & \times {\left\{(1-\gamma ){\left[h_{0-}(y_{-i})\text{exp}(\lambda_{-}z_{-i}+\sum_{j=2}^{k}\beta_{-j}z_{-j})\right]}^{\delta_{-i}}\text{exp}\left[-H_{0-}\text{exp}(\lambda_{-}z_{-i}+\sum_{j=2}^{k}\beta_{-j}z_{-j})\right]\right\}}^{1-x_{i}} \end{array}$$

where z_*j*_ and *β*_*j*_ are the other covariates and their corresponding regression coefficients.

Similarly, the regression coefficients can be estimated by the EM algorithm and their estimated standard errors can be obtained via the bootstrap technique.

Since no diagnostic tool is perfect with 100% PPV, our proposed EM procedure is based on the Cox PH model given two latent class memberships. However, we would be interested in the impact of violation of the two latent classes’ assumption. An additional simulation study was conducted to assess the performance for the special case of PPV = 1.00. The simulation results on the relative bias of the estimator for hazard ratio by both EM and naïve methods are provided in Table 6. As expected, the naïve method performs slightly better than the EM procedure when a prefect diagnostic tool is available. In such a situation if the PPV is 100%, we suggest the use of the naïve method. We also note that relative biases by both methods do not exceed 5%. The case of PPV = 1.00 has little impact on the bias of the EM procedure. On the other hand, the simulation results reveal that differences between the naïve method and the EM procedure decrease when the PPV increases.

**Table 6:** Relative bias (%) for the case of PPV = 1.00 at n = 300 per group.

HR										
	0.65		0.70		0.75		0.80		0.85	
CR	Naive	EM	Naive	EM	Naive	EM	Naive	EM	Naive	EM
0.3	3.13	4.38	3.21	4.65	2.90	3.93	3.07	4.08	2.88	3.95
0.4	3.61	4.92	3.55	4.88	3.26	4.53	3.40	4.87	3.48	4.79

CR: censored rate; HR: hazard ratio

We conducted another simulation study to investigate the robustness of our proposed EM procedure when the true-positive data is generated from the Weibull distribution with a shape parameter of 2 and the false-positive data is from an exponential distribution. Table 7 shows the relative bias in estimating the hazard ratio when n = 300, the censoring rate = 30%, and the hazard ratio is 0.75 with the PPV ranging from 0.5 to 0.9 in increments of 0.1. It appears that under these conditions the naïve method performs slightly better than the EM procedure based on the Cox PH model. This indicates that violation of the distribution assumption can lead to a biased estimate of the hazard ratios. To remedy such a situation, future work will extend our proposed EM procedure to proportional hazards models using the parametric Weibull model, which has hazard function

$$h(t|z)=pt^{p-1}\text{exp}(\lambda z),$$

for parameter *p*>0.

**Table 7:** Relative bias (%) for the Weibull simulation model at n = 300 per group, HR = 0.75, and 0.3 of censored rate.

Method	PPV				
	0.5	0.6	0.7	0.8	0.9
Naive	11.63	11.12	13.45	18.12	25.69
EM	17.45	16.35	18.49	20.89	26.98

## Supporting Information

S1 Fig Relative bias curves between the proposed EM and naive approach for different censored rates (CR) at sample size n = 600 per group. Black line: naive; red line: proposed EM.
(PDF)

S2 Fig Relative bias curves between the proposed EM and naive approach for different censored rates (CR) at sample size n = 900 per group. Black line: naive; red line: proposed EM.
(PDF)

S3 Fig Empirical power curves between the proposed EM and naive approach for different censored rates (CR) at sample size n = 600 per group. Black line: naive; red line: proposed EM.
(PDF)

S4 Fig The empirical power curve between EM approach and naive approach for different censored rates (CR) at sample size n = 900 per group. Black line: naive; red line: proposed EM.
(PDF)

S1 Table Relative bias (%) and coverage probability for n = 600 per group. (PDF)

S2 Table Relative bias (%) and coverage probability for n = 900 per group. (PDF)

S3 Table Comparison of empirical powers for n = 600 per group. (PDF)

S4 Table Comparison of empirical powers for n = 900 per group. (PDF)
