# Supplementary material for: Addressing Loss of Efficiency Due to Misclassification Error in Enriched Clinical Trials for the Evaluation of Targeted Therapies Based on the Cox Proportional Hazards Model
Source: PLoS One. 2016 Apr 27;11(4):e0153525. doi: 10.1371/journal.pone.0153525 (PMC4847784; doi:10.1371/journal.pone.0153525)
Supplement: S4 Fig — Black line: naive; red line: proposed EM. (PDF) [file pone.0153525.s004.pdf]

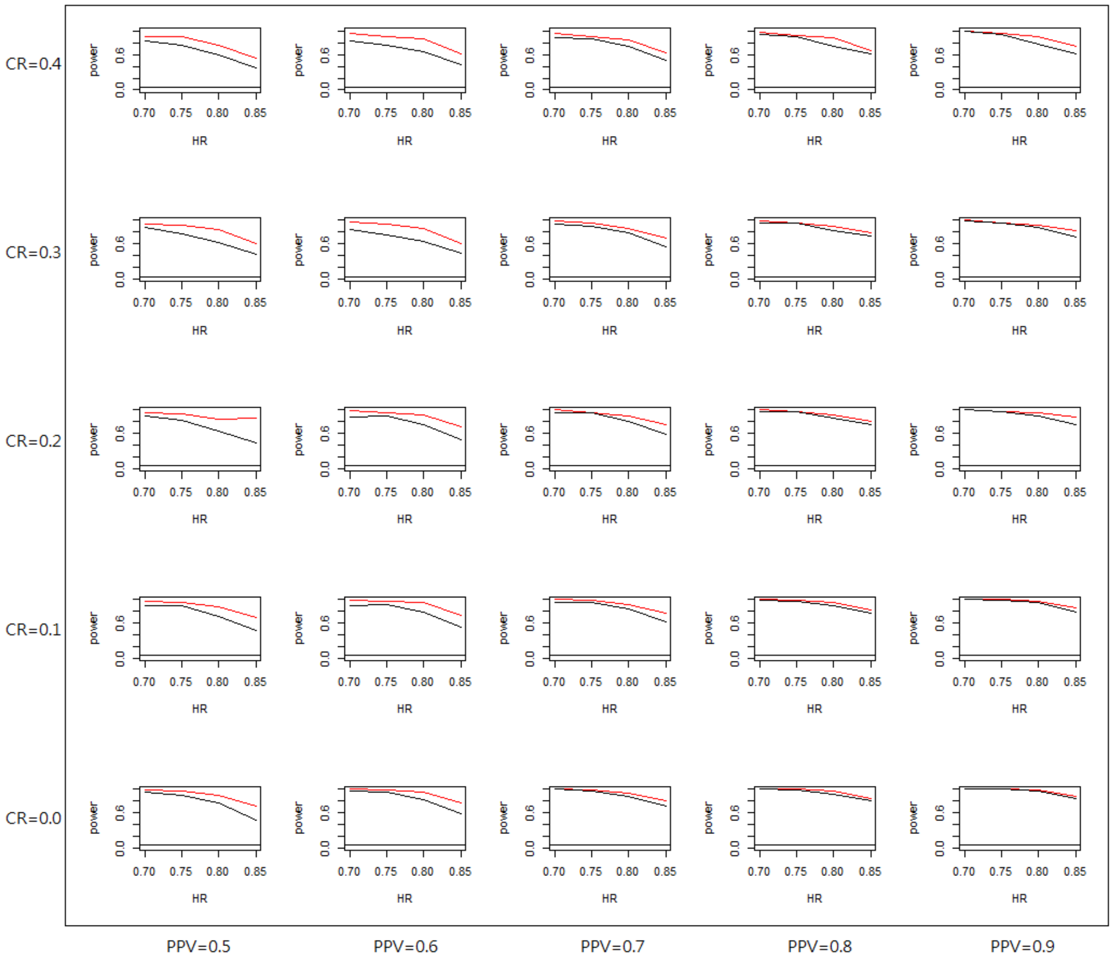

Figure S4 The empirical power curve between EM approach and naive approach for different censored rates (CR) at sample size n=900 per group. black line: naive; red line: proposed EM
