# Supplementary material for: Addressing Loss of Efficiency Due to Misclassification Error in Enriched Clinical Trials for the Evaluation of Targeted Therapies Based on the Cox Proportional Hazards Model
Source: PLoS One. 2016 Apr 27;11(4):e0153525. doi: 10.1371/journal.pone.0153525 (PMC4847784; doi:10.1371/journal.pone.0153525)
Supplement: S1 Table — (PDF) [file pone.0153525.s005.pdf]

Table S1 Relative bias (%) and coverage probability for n=600 per group

			PPV									
			0.5 (0.501)		0.6 (0.598[c])		0.7 (0.694[c])		0.8 (0.792[c])		0.9 (0.902[c])	
n	HR	CR	Naive	EM	Naive	EM	Naive	EM	Naive	EM	Naive	EM
600	0.85	0	9.05[a]	1.05	6.58	-0.25	5.40	0.46	4.34	1.52	1.28	0.11
			0.782[b]	0.966	0.815	0.959	0.881	0.955	0.903	0.953	0.933	0.963
		0.1	8.69	1.01	6.82	0.42	5.52	0.58	4.38	1.48	1.86	0.38
			0.798	0.945	0.814	0.942	0.898	0.963	0.917	0.948	0.932	0.953
		0.2	9.28	0.81	7.28	0.58	5.75	0.80	4.11	1.40	2.22	0.45
			0.803	0.930	0.810	0.950	0.891	0.946	0.901	0.943	0.925	0.950
		0.3	10.34	0.57	7.16	0.23	5.75	0.22	4.46	1.04	1.40	0.13
			0.800	0.930	0.808	0.936	0.880	0.939	0.909	0.951	0.923	0.951
		0.4	8.46	-0.13	6.69	-0.13	5.64	0.58	4.22	1.40	3.28	1.87
			0.796	0.927	0.804	0.933	0.887	0.949	0.913	0.958	0.939	0.966
	0.8	0	11.48	0.73	9.11	0.61	7.23	1.11	4.48	0.86	2.98	1.11
			0.745	0.954	0.784	0.946	0.872	0.956	0.910	0.958	0.931	0.958
		0.1	11.48	0.78	9.11	0.58	7.11	0.26	4.61	0.48	2.23	0.23
			0.753	0.941	0.769	0.962	0.874	0.951	0.908	0.953	0.928	0.954
		0.2	12.23	0.98	10.48	0.61	7.36	0.23	4.83	0.48	2.98	0.61
			0.760	0.933	0.773	0.936	0.855	0.931	0.904	0.940	0.928	0.949
		0.3	11.86	-0.51	9.86	-0.13	7.13	-0.26	5.23	1.11	2.72	0.86
			0.772	0.932	0.792	0.939	0.874	0.938	0.900	0.937	0.923	0.943
		0.4	12.23	0.23	10.86	1.48	7.23	-0.38	5.48	1.61	3.33	1.61
			0.775	0.933	0.801	0.926	0.872	0.937	0.889	0.943	0.931	0.950

a: Relative bias (%)   b: Coverage probability   c:Estimate of PPV

CR: censored rate   HR: hazard ratio

Table S1 Relative bias (%) and coverage probability for n=600 per group (continued)

			PPV									
			0.5 (0.501)		0.6 (0.598[c])		0.7 (0.694[c])		0.8 (0.792[c])		0.9 (0.902[c])	
n	HR	CR	Naive	EM	Naive	EM	Naive	EM	Naive	EM	Naive	EM
600	0.75	0	14.92[a]	0.78	11.85	0.92	9.45	1.05	5.85	0.78	3.05	0.52
			0.684[b]	0.957	0.753	0.949	0.847	0.960	0.898	0.959	0.922	0.951
		0.1	15.05	-0.54	12.11	0.25	9.58	0.52	6.12	0.52	2.52	-0.23
			0.702	0.941	0.764	0.946	0.861	0.953	0.907	0.954	0.931	0.958
		0.2	15.05	-1.48	12.12	-0.14	9.44	-0.26	5.44	-0.67	2.78	-0.41
			0.722	0.936	0.766	0.930	0.852	0.943	0.908	0.953	0.921	0.946
		0.3	15.18	-1.61	12.10	-0.67	9.18	-0.41	6.78	0.92	2.91	-1.21
			0.736	0.931	0.779	0.928	0.863	0.941	0.910	0.947	0.916	0.946
		0.4	15.32	-0.14	12.51	0.12	9.45	-0.14	5.18	0.12	2.78	0.25
			0.755	0.926	0.779	0.934	0.860	0.931	0.924	0.961	0.909	0.947
	0.7	0	18.98	1.55	14.98	0.70	10.55	0.55	6.98	0.55	4.12	0.98
			0.618	0.942	0.688	0.946	0.832	0.956	0.889	0.944	0.923	0.946
		0.1	19.84	-0.29	14.93	-0.44	10.84	-0.15	7.26	0.41	4.41	0.82
			0.638	0.946	0.703	0.937	0.836	0.943	0.895	0.947	0.931	0.958
		0.2	19.11	-1.01	15.12	-1.01	10.70	-1.01	7.71	0.41	4.12	0.70
			0.661	0.937	0.725	0.936	0.844	0.942	0.895	0.938	0.921	0.941
		0.3	19.27	-1.15	15.12	-0.87	10.69	-1.58	7.41	0.12	3.28	-0.15
			0.683	0.936	0.727	0.939	0.846	0.938	0.893	0.928	0.945	0.969
		0.4	19.41	-0.72	15.97	0.55	10.70	-0.72	7.98	1.12	4.10	1.14
			0.695	0.941	0.726	0.941	0.855	0.937	0.917	0.950	0.919	0.947

a: Relative bias (%)   b: Coverage probability   c: Estimate of PPV
CR: censored rate   HR: hazard ratio
