# Supplementary material for: Addressing Loss of Efficiency Due to Misclassification Error in Enriched Clinical Trials for the Evaluation of Targeted Therapies Based on the Cox Proportional Hazards Model
Source: PLoS One. 2016 Apr 27;11(4):e0153525. doi: 10.1371/journal.pone.0153525 (PMC4847784; doi:10.1371/journal.pone.0153525)
Supplement: S3 Table — (PDF) [file pone.0153525.s007.pdf]

Table S3 Comparison of empirical powers for n=600 per group

			PPV									
			0.5		0.6		0.7		0.8		0.9	
n	HR	CR	Naive	EM	Naive	EM	Naive	EM	Naive	EM	Naive	EM
600	0.85	0	0.336	0.526	0.425	0.576	0.505	0.615	0.544	0.615	0.575	0.617
		0.1	0.335	0.510	0.388	0.560	0.450	0.583	0.530	0.600	0.545	0.613
		0.2	0.314	0.486	0.361	0.543	0.422	0.581	0.511	0.601	0.519	0.630
		0.3	0.299	0.448	0.339	0.498	0.399	0.538	0.506	0.588	0.490	0.593
		0.4	0.267	0.396	0.321	0.467	0.360	0.489	0.422	0.490	0.422	0.532
	0.8	0	0.571	0.775	0.629	0.796	0.718	0.811	0.775	0.822	0.799	0.827
		0.1	0.531	0.765	0.602	0.795	0.685	0.800	0.762	0.802	0.777	0.820
		0.2	0.479	0.738	0.582	0.769	0.659	0.787	0.736	0.787	0.743	0.798
		0.3	0.468	0.736	0.540	0.739	0.655	0.756	0.713	0.770	0.729	0.772
		0.4	0.446	0.667	0.499	0.728	0.616	0.750	0.643	0.758	0.646	0.765
	0.75	0	0.747	0.911	0.825	0.927	0.841	0.936	0.876	0.951	0.948	0.961
		0.1	0.747	0.902	0.791	0.911	0.830	0.928	0.866	0.933	0.936	0.950
		0.2	0.687	0.894	0.784	0.902	0.822	0.911	0.866	0.916	0.915	0.920
		0.3	0.649	0.874	0.717	0.895	0.776	0.909	0.858	0.912	0.912	0.914
		0.4	0.632	0.862	0.656	0.861	0.756	0.867	0.820	0.886	0.902	0.911
	0.7	0	0.815	0.959	0.933	0.972	0.941	0.991	0.968	0.993	0.981	1.000
		0.1	0.781	0.932	0.873	0.964	0.890	0.984	0.923	0.987	0.980	0.989
		0.2	0.779	0.924	0.849	0.961	0.889	0.969	0.890	0.963	0.954	0.975
		0.3	0.765	0.914	0.821	0.946	0.878	0.952	0.888	0.964	0.963	0.968
		0.4	0.723	0.883	0.808	0.928	0.833	0.933	0.852	0.934	0.926	0.938

CR: censoring rate HR: hazard ratio
