# Supplementary material for: Addressing Loss of Efficiency Due to Misclassification Error in Enriched Clinical Trials for the Evaluation of Targeted Therapies Based on the Cox Proportional Hazards Model
Source: PLoS One. 2016 Apr 27;11(4):e0153525. doi: 10.1371/journal.pone.0153525 (PMC4847784; doi:10.1371/journal.pone.0153525)
Supplement: S4 Table — (PDF) [file pone.0153525.s008.pdf]

Table S4 Comparison of empirical powers for n=900 per group

			PPV									
			0.5		0.6		0.7		0.8		0.9	
n	HR	CR	Naive	EM	Naive	EM	Naive	EM	Naive	EM	Naive	EM
900	0.85	0	0.471	0.710	0.578	0.755	0.697	0.799	0.789	0.830	0.833	0.864
		0.1	0.469	0.688	0.527	0.734	0.621	0.757	0.769	0.811	0.791	0.858
		0.2	0.439	0.656	0.490	0.712	0.582	0.756	0.742	0.812	0.753	0.882
		0.3	0.419	0.605	0.461	0.652	0.550	0.699	0.734	0.794	0.711	0.830
		0.4	0.374	0.535	0.437	0.612	0.497	0.635	0.611	0.662	0.611	0.745
	0.8	0	0.765	0.884	0.818	0.947	0.869	0.925	0.899	0.962	0.967	0.975
		0.1	0.712	0.872	0.783	0.946	0.829	0.912	0.883	0.939	0.940	0.967
		0.2	0.642	0.841	0.757	0.915	0.798	0.897	0.854	0.921	0.899	0.942
		0.3	0.627	0.839	0.702	0.880	0.793	0.862	0.828	0.901	0.883	0.911
		0.4	0.598	0.760	0.648	0.866	0.745	0.855	0.746	0.887	0.782	0.903
	0.75	0	0.896	0.957	0.949	0.973	0.967	0.983	0.973	0.996	0.996	0.999
		0.1	0.896	0.947	0.910	0.957	0.955	0.974	0.962	0.978	0.983	0.997
		0.2	0.825	0.939	0.902	0.947	0.946	0.957	0.961	0.960	0.961	0.966
		0.3	0.779	0.918	0.824	0.940	0.892	0.955	0.952	0.956	0.958	0.960
		0.4	0.758	0.905	0.755	0.904	0.869	0.911	0.910	0.929	0.947	0.957
	0.7	0	0.937	0.987	0.961	0.997	0.997	1.000	1.000	1.000	1.000	1.000
		0.1	0.898	0.960	0.900	0.989	0.943	1.000	0.988	1.000	1.000	1.000
		0.2	0.896	0.952	0.875	0.986	0.942	0.998	0.967	0.996	1.000	1.000
		0.3	0.879	0.941	0.846	0.971	0.931	0.981	0.950	0.997	0.999	1.000
		0.4	0.831	0.909	0.833	0.952	0.883	0.961	0.936	0.967	0.997	1.000

CR: censoring rate   HR: hazard ratio
